# Prediction of metabolic syndrome and its associated risk factors in patients with chronic kidney disease using machine learning techniques

**DOI:** 10.1590/2175-8239-JBN-2023-0135en

**Published:** 2024-08-09

**Authors:** Jalila Andréa Sampaio Bittencourt, Carlos Magno Sousa, Ewaldo Eder Carvalho Santana, Yuri Armin Crispim de Moraes, Erika Cristina Ribeiro de Lima Carneiro, Ariadna Jansen Campos Fontes, Lucas Almeida das Chagas, Naruna Aritana Costa Melo, Cindy Lima Pereira, Margareth Costa Penha, Nilviane Pires, Edward Araujo, Allan Kardec Duailibe Barros, Maria do Desterro Soares Brandão Nascimento

**Affiliations:** 1 Universidade Federal do Maranhão, Departamento de Engenharia Eletrônica, Laboratório de Processamento da Informação Biológica, São Luiz, MA, Brazil.; 2 Universidade Federal do Maranhão, Departamento de Ciência da Computação, Laboratório de Aquisição e Processamento de Sinais, São Luiz, MA, Brazil.; 3 Universidade Federal do Maranhão, Hospital Universitário, Centro de Prevenção de Doenças Renais, São Luiz, MA, Brazil.; 4 Universidade Federal do Maranhão, Centro de Ciências Biológicas e da Saúde, Laboratório de Imunofisiologia, São Luiz, MA, Brazil.; 5 Universidade Federal de São Paulo, Escola Paulista de Medicina, Departamento de Obstetrícia, São Paulo, SP, Brazil.; 6 Universidade Federal do Maranhão, Laboratório de Ciências Biológicas, Laboratório de Genética e Biologia Molecular, São Luiz, MA, Brazil.; 7 Universidade Ceuma, Departamento de Biomedicina, Laboratório de Ciências Biomédicas, São Luiz, MA, Brazil.; 8 Universidade Federal do Maranhão, Departamento de Medicina, Programa de Pós-Graduação em Saúde do Adulto, São Luiz, MA, Brazil.

**Keywords:** Artificial Intelligence, Renal Insufficiency, Chronic, Machine Learning, Metabolic syndrome

## Abstract

**Introduction:**

Chronic kidney disease (CKD) and metabolic syndrome (MS) are recognized as public health problems which are related to overweight and cardiometabolic factors. The aim of this study was to develop a model to predict MS in people with CKD.

**Methods:**

This was a prospective cross-sectional study of patients from a reference center in São Luís, MA, Brazil. The sample included adult volunteers classified according to the presence of mild or severe CKD. For MS tracking, the k-nearest neighbors (KNN) classifier algorithm was used with the following inputs: gender, smoking, neck circumference, and waist-to-hip ratio. Results were considered significant at p < 0.05.

**Results:**

A total of 196 adult patients were evaluated with a mean age of 44.73 years, 71.9% female, 69.4% overweight, and 12.24% with CKD. Of the latter, 45.8% had MS, the majority had up to 3 altered metabolic components, and the group with CKD showed statistical significance in: waist circumference, systolic blood pressure, diastolic blood pressure, and fasting blood glucose. The KNN algorithm proved to be a good predictor for MS screening with 79% accuracy and sensitivity and 80% specificity (area under the ROC curve – AUC = 0.79).

**Conclusion:**

The KNN algorithm can be used as a low-cost screening method to evaluate the presence of MS in people with CKD.

## Introduction

Chronic non-communicable diseases (CNCD) are currently recognized as one of the major public health problems^1^. The World Health Organization (WHO) estimates that CNCDs are responsible for 71% of the 57 million deaths worldwide^2^. In Brazil, CNCDs are responsible for 76.4% of all deaths, with a focus on diseases of the circulatory system (28% of deaths), cancer (18%), diabetes mellitus (5%), and respiratory diseases (6%)^3^.

Among the CNCD, chronic kidney disease (CKD), which is characterized by altered renal function, stands out. It is defined as an abnormality in renal structure or function that has been present for more than three months and has health implications. These abnormalities can be represented by a decreased glomerular filtration rate (GFR) <60 ml/min/1.73 m or the presence of one or more markers of kidney injury^4,5^.

The prevalence of CKD is still unknown in many countries^6^. However, it has been increasing, mainly as a result of the increasing incidence of obesity, diabetes, and hypertension. In addition, renal function is highly susceptible to age-related changes, with a significantly higher incidence in middle-aged and elderly patients^7,8^.

People with CKD tend to have cardiovascular disease (CVD)^8^. CVD is the leading cause of death in patients with chronic kidney disease and is associated with accelerated progression of CKD. These findings support the view that the presence of cardiometabolic risk factors (CRF) and impaired kidney function may increase kidney disease-related risks^9^.

In addition, metabolic syndrome (MS) is considered to be a grouping of interrelated risk factors that doubles the risk of CVD in 5 to 10 years^10^. This pathology is described as a set of the CRF, which are usually related to the development of insulin resistance and fat accumulation. These risk factors include arterial hypertension, hypertriglyceridemia, dyslipidemia, hyperglycemia and central obesity^11^.

Individuals with NCD generate great financial costs to the public health system as they require treatment to control these diseases, especially in MS and CKD. Patients with end-stage CKD, stage G5 (GFR <15 mL/min/1.73 m^2^) have severe renal failure leading to complete loss of renal function. At this stage, the therapeutic options are renal replacement therapies (RRT), such as artificial blood purification methods (peritoneal dialysis or hemodialysis) or kidney transplantation^4^. In Brazil, RRT is considered the main treatment and also requires higher costs for health services^12^. Therefore, early detection of such pathologies can delay complications and support the use of appropriate interventions, such as screening tests in high-risk groups^13^.

In this case, some data analysis techniques appear to be good solutions that provide more accurate predictions about the individual’s health^14^. Therefore, the use of machine learning (ML) techniques appears to be an instrument to help develop and improve new methods for diagnosis and/or screening^15,16^.

Therefore, the importance of the study for the prediction of MS in the population with CKD is clear, since this condition leads to more advanced stages and a higher risk of death from cardiovascular events in this population. Therefore, even though patients with CKD have few risk factors for MS, preventive measures must be taken to avoid problems and negative outcomes such as early death.

Thus, given the magnitude of MS and CKD and the complications related to them, efforts should therefore be made to enable studies aimed at early diagnosis of these pathologies. In addition, this study aims to develop a model to predict the risk of MS and associated risk factors in people with CKD.

## Methods

This was a prospective cross-sectional study of patients of both sexes from the Nephrology Reference Center in São Luís, MA, Brazil, between January 2018 and July 2020. The sample consisted of 196 volunteers classified according to their health status. CKD was determined by a glomerular filtration rate <60 mL/min^4^, for mild CKD (above this value) or severe CKD. GFR was determined by measuring serum creatinine To calculate the estimated glomerular filtration rate, the Chronic Kidney Disease Epidemiology Collaboration (CKD-EPI) equation was used^17^.

The suspected diagnosis of MS was defined as proposed by the International Diabetes Federation (IDF)^18^ by considering the presence of changes in waist circumference (≥ 90 cm for men and ≥ 80 cm for women), as a mandatory factor in addition to two other altered components. These components may be: triglycerides ≥ 150 mg/dL or treatment for dyslipidemia; HDL cholesterol < 40 mg/dL for men and < 50 mg/dL for women or treatment for dyslipidemia; systolic blood pressure (SBP) ≥ 130 mmHg, diastolic blood pressure (DBP) ≥ 85 mmHg, or use of antihypertensive medication; and fasting blood glucose ≥ 100 mg/dL or previous diagnosis of diabetes mellitus^19^.

Data collection was carried out in two stages. First, volunteers were recruited at the Nephrology Center. Those who agreed to participate in the study voluntarily signed the Informed Consent Form (ICF). Soon after, they were interviewed using a semi-structured questionnaire, followed by an anthropometric and hemodynamic assessment. The laboratory tests were then scheduled for the following day.

Anthropometric, biochemical, hemodynamic, and lifestyle data were evaluated. The semi-structured questionnaire took into account sociodemographic characteristics, lifestyle, and self-reported personal history, such as hypertension and diabetes mellitus. Anthropometric variables were performed in duplicate, and the means were used for data analysis. All variables were measured according to protocols already consolidated in the literature^20^. These variables were: weight, height, arm circumference (AC), waist circumference (WC), hip circumference (HC), neck circumference (NC), and calf circumference (CC).

Anthropometric indices: waist-to-hip ratio (WHR), waist-to-height ratio (WHtR), and body mass index (BMI) were used to determine nutritional status and were based on WHO cutoff points. WC was estimated considering the cutoff points for the South American population, with values of ≥90 cm for men and ≥80 cm for women^19^.

Only the k-nearest neighbors (KNN) classifier algorithm, a supervised ML method, was used for MS tracking. It is easy to implement, adaptable, and easy to program. All these advantages had a positive impact on our choice, as KNN alone met our demands^21^.

KNN uses the closest data and performs a segmentation of the closest results based on the selected metric by considering a limited margin of error. In this algorithm, the dataset is prepared by removing missing values and normalizing features, which is known as the pre-processing phase. The data is randomly divided into two different sets: the training set and the test set. This technique ensures an adequate representation of the patterns for training and a robust performance evaluation^21^.

Therefore, the database was divided into two groups: 80% of the sample was for training and 20% for testing. Based on the patient’s clinical information (gender, smoking, WHR, and CC), the classifier algorithm was able to distinguish individuals with and without MS. The classification method was constructed using MATLAB^®^ software version R2021a (MathWorks Inc, Natick, MD, USA).

For the data file and the statistical analysis, the SPSS^®^ software version 25 (SPSS Inc., Chicago, IL, USA) was used. The Kolmogorov-Smirnov test was used to analyze the normality of the data. The variables that were considered normally distributed were analyzed using the Student’s t-test. The others were analyzed with the Mann-Whitney U test, and the Receiver Operating Characteristics (ROC) curve was used for the evaluation of the algorithm classifier. In addition, the results were considered statistically significant at p < 0.05. All statistical analysis values are described in the tables in the Results section.

This study is part of a larger project (“umbrella” or “root”), entitled “Prediction of Chronic Kidney Disease Using Artificial Neural Networks”. It was also approved by the Ethics and Research Committee of the Federal University of Maranhão, according to the CAAE opinion number 67030517.5.0000.5087. In addition, all participants signed the informed consent form. It is worth mentioning that this work uses original population data generated exclusively for this research, in addition to providing new data for the umbrella project. Furthermore, it is worth mentioning that both neural networks and the KNN are artificial intelligence computational models that work with data processing for classification and prediction.

## Results

A total of 196 adult patients with a mean age of 44.73 ± 15.96 years were evaluated, of whom 71.9% (n = 141) were female and 79.1% (n = 155) identified themselves as non-white. In addition, 65.8% (n = 129) of patients reported no physical activity, 29.1% (n = 57) consumed alcohol, and 3.6% (n = 7) smoked. Regarding underlying diseases, 31.1% (n = 61) reported having systemic arterial hypertension (SAH) and 29.6% (n = 58) had diabetes mellitus (Table 1). The analysis of anthropometric data revealed that more than half of the individuals were overweight (69.4%), with a mean BMI of 27.44 ± 5.10 kg/m^2^ (Tables 1 and 2).

**Table 1:** Sociodemographic and lifestyle characteristics of the sample

Variables	Category	N	%
Skin color^*^	White	41	20.9%
	Not white	155	79.1%
Gender^*^	Women	141	71.9%
	Man	55	28.1%
Nutritional status (BMI)^*^	Eutrophic	60	30.6%
	Overweight	136	69.4%
Smoking^*^	No	189	96.4%
	Yes	7	3.6%
Alcoholism^*^	No	139	70.9%
	Yes	57	29.1%
Physical activity practice^*^	No	129	65.8%
	Yes	67	34.2%
Presence of CKD^*^	No	172	87.8%
	Yes	24	12.2%
Presence of MS^*^	No	150	76.5%
	Yes	46	23.5%
DM self-reported^*^	No	138	70.4%
	Yes	58	29.6%
Self-reported SAH^*^	No	135	68.9%
	Yes	61	31.1%

Abbreviations – BMI: body mass index; CKD: chronic kidney disease; MS: metabolic syndrome; DM: diabetes mellitus; SAH: systemic arterial hypertension. Notes – ^*^values are described as percentage (%) and frequency (n).

**Table 2:** Anthropometric and hemodynamic characteristics of the sample

Variables	Averages
Age (years)^†^	44.73 ± 15.96
Height (m)^†^	1.58 ± 0.08
Body weight (kg)^†^	68.63 ± 13.79
WC (cm)^†^	87.22 ± 12.95
AC (cm)^†^	30.46 ± 4.08
NC (cm)^†^	35.16 ± 3.53
CC (cm)^†^	35.66 ± 3.63
BMI (kg/m^2^)^†^	27.44 ± 5.10
WHtR^†^	0.55 ± 0.08
WHR^†^	0.85 ± 0.08
SBP^†^	124.06 ± 23.99
DBP^†^	77.37 ± 12.06

Abbreviations – WC: waist circumference; AC: Arm circumference; NC: neck circumference; CC: calf circumference; BMI: body mass index; WHtR: waist to height ratio; WHR: waist: hip ratio; SBP: systolic blood pressure; DBP: diastolic blood pressure; m: meter; cm: centimeter. Notes – ^†^Mann-Whitney U test; Data are described as mean ± (standard deviation).

Table 3 shows the general characteristics of the sample, which was stratified according to the presence of mild and severe CKD. In this Table, the severe CKD patients had significantly higher values of systolic and diastolic blood pressure, age, CP, fasting blood glucose, and urea (p < 0.05) than those with mild CKD. Similarly, the obesity indicator variables (WC, BMI, WHR, WHtR) also showed a higher prevalence in the severe CKD group.

**Table 3:** Anthropometric, hemodynamic, and laboratory characteristics of the sample, stratified by the presence of chronic kidney disease (CKD)

Variables	Mild CKD	Severe CKD	p-value
	(n = 172)	(n = 24)	
Age (years)^†^	40.5 (29.25 – 54)	60 (51.25 – 68)	<0.001
Height (m)^†^	1.57 (1.51 – 1.63)	1.61 (1.55 – 1.68)	0.153
Body weight (kg)^†^	66.2 (58.8 – 76.57)	70.45 (64.17 – 80.45)	0.090
WC (cm)^†^	86.16 ± 12.79	94.85 ± 11.7	0.002
AC (cm)^†^	30 (28 – 33.3)	30 (28.12 – 31.87)	0.816
NC (cm)^†^	34.9 (32 – 38)	36.75 (34 – 39.75)	0.039
CC (cm)^†^	35.5 (33 – 38)	34.25 (32 – 36.75)	0.196
BMI (kg/m^2^)^†^	26.54 (23.71 – 29.91)	27.67 (25.58 – 31.42)	0.285
WHR^†b^	0.84 ± 0.08	0.93 ± 0.08	<0.001
WHtR^†^	0.54 ± 0.08	0.59 ± 0.07	0.012
SBP^†^	117.5 (107 – 131)	134 (123.25 – 165.5)	<0.001
DBP^†^	75 (70 – 82)	86.5 (76.25 – 91)	0.019
TC^†^	178.12 ± 45.39	163.13 ± 44.78	0.131
Triglycerides^†^	108 (75.25 – 164.75)	128 (84.25 – 174.5)	0.216
HDL-cholesterol^†^	44 (38 – 54)	43 (34 – 53.75)	0.575
LDL-cholesterol^†^	105.73 ± 36.41	89.80 ± 41.89	0.050
Blood glucose^†^	79.00 (77.50 – 87.50)	81.00 (75.00 – 88.00)	0.001
Urea^†^	24.5 (20.25 – 31)	54.5 (39.25 – 79)	<0.001

Abbreviations – BMI: body mass index; WC: waist circumference; AC: Arm circumference; CC: calf circumference; WHR: waist:hip ratio; WHtR: waist to height ratio; SBP: systolic blood pressure; DBP: diastolic blood pressure; TC: total cholesterol; GFR: glomerular filtration rate; m: meter; cm: centimeter. Notes – ^†^Mann-Whitney U test; Values are described as mean/median ± SD (standard deviation).

For clinical, epidemiological, didactic, and conceptual purposes, CKD is classified into six functional stages according to the patient’s degree of renal function based on glomerular filtration rate, ranging from normal/high condition to dialysis or transplantation. The Kidney Disease: Improving Global Outcomes (KDIGO) guides the estimation of GFR from serum creatinine as the best method to diagnose, classify and monitor progression of CKD^4^. GFR was categorized from G1 to G5 as shown in Table 4.

**Table 4:** Staging and classification of chronic kidney disease (CKD) in the sample (N = 196)

Stages	Classification		Frequency^*^	
G1	TFG >90	(normal or increased)	117	41.1%
G2	TFG 60–89	(slightly diminished)	55	19.3%
G3a	TFG 45–59	(mild to moderate decrease)	10	3.5%
G3b	TFG 30–44	(moderate to severe decrease)	5	1.8%
G4	TFG 15–29	(severely diminished)	5	1.8%
G5	TFG <15	(renal insufficiency)	4	1.4%

Abbreviation – GFR: glomerular filtration rate. Notes – ^*^values are described in frequency (n) and percentage (%).

Regarding the staging and classification of CKD, Table 4 shows that a large part of the sample is in the initial stages (G1 and G2), all from the mild CKD group. In contrast, only 1.4% (n = 4) is in the end-stage and requires some type of renal replacement therapy.

Table 5 shows the prevalence values of risk factors for metabolic syndrome stratified by the presence of mild or severe CKD. The analysis of clinical data showed that, in percentage values, all altered components of MS were found to be prevalent in the severe CKD group, with the exception of total cholesterol (TC). Of the investigated sample, 45.8% of patients with severe CKD had MS, and the majority had up to 3 altered metabolic components.

**Table 5:** Prevalence of metabolic syndrome and its components in the sample

Variables	Category	Mild CKD		Severe CKD		p-value
		N	%	N	%	
MS^*^	Not	137	79.70%	13	54.20%	0.006
	Yes	35	20.30%	11	45.80%
WC^*^	Normal	68	39.50%	6	25.00%	0.169
	Changed	104	60.50%	18	75,00%
BP^*^	Normal	113	65.70%	7	29.20%	0.001
	Changed	59	34.30%	17	70.80%
TC^*^	Normal	108	62.80%	17	70,80%	0.443
	Changed	64	37.20%	7	29.20%
TG^*^	Normal	121	70.30%	15	62.50%	0.434
	Changed	51	29.70%	9	37.50%
HDL^*^	Not	75	43.60%	10	41.70%	0.858
	Yes	97	56.40%	14	58.30%
FG^*^	Normal	149	86.60%	15	62.50%	0.003
	Changed	23	13.40%	9	37.50%

Abbreviations – WC: waist circumference; BP: blood pressure; high TC: high total cholesterol; TG: triglycerides; HDL: high density lipoprotein; GJ: fasting blood glucose. Note – ^*^Chi-square test.

When we look at the table stratified by the presence of CKD (Table 3), the group of individuals with kidney problems had higher mean/median values in all parameterscompared to healthy individuals, with statistical significance in WC, systolic blood pressure, diastolic blood pressure and fasting blood glucose.

Regarding the software developed through ML, the implemented algorithm was the KNN with the following entries: gender, smoking, WHR, and NC. The development of the algorithm was labeled based on the MS components according to the IDF criteria^18,19^.

There is no fixed ratio that works in all scenarios in the KNN algorithm. The database was divided into two groups: 80% of the sample was allocated for training and 20% for testing. This was done taking into account the fact that, with very large datasets, it may be feasible to use ratios such as 70/30 or even 90/10^22^. All these divisions were tested, and the one that showed the best response was 80/20, as it is a medium sized database. The KNN had 79% accuracy and sensitivity and a specificity of 80%.

**Figure 1.**
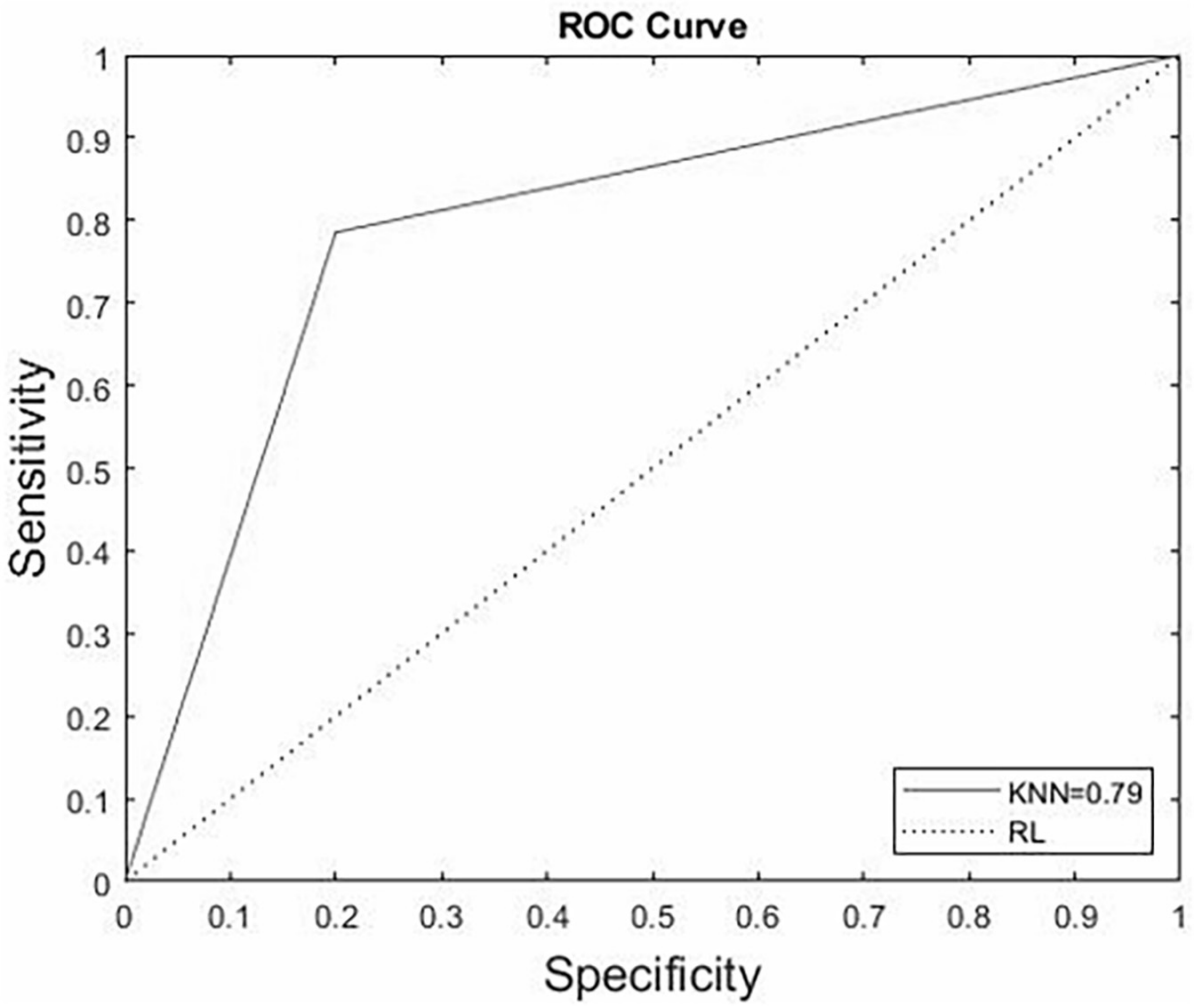
Area under the ROC curve is demonstrating the discriminatory power of the k-nearest neighbors’ algorithm in predicting metabolic syndrome in the test set.

Figure 1 shows the graphical representation of the ROC curve (area under the ROC curve – AUC = 0.79) generated by plotting the sensitivity (true positive rate) on the y-axis against the specificity (false positive rate) on the x-axis. Thus, the KNN proved to be a good classifier for predicting MS. For a diagnostic test to be considered accurate, a curve in the upper left triangle must be above the reference line. The closer this curve is to this corner, i.e. closer to 1, the better the method’s performance^23,24^.

In this first stage of the study, we decided to use these anthropometric input parameters as they are inexpensive, easy to use and already recommended in the literature. However, it is believed that using more input variables can increase the specificity of the classifier algorithm.

**Figure 2:**
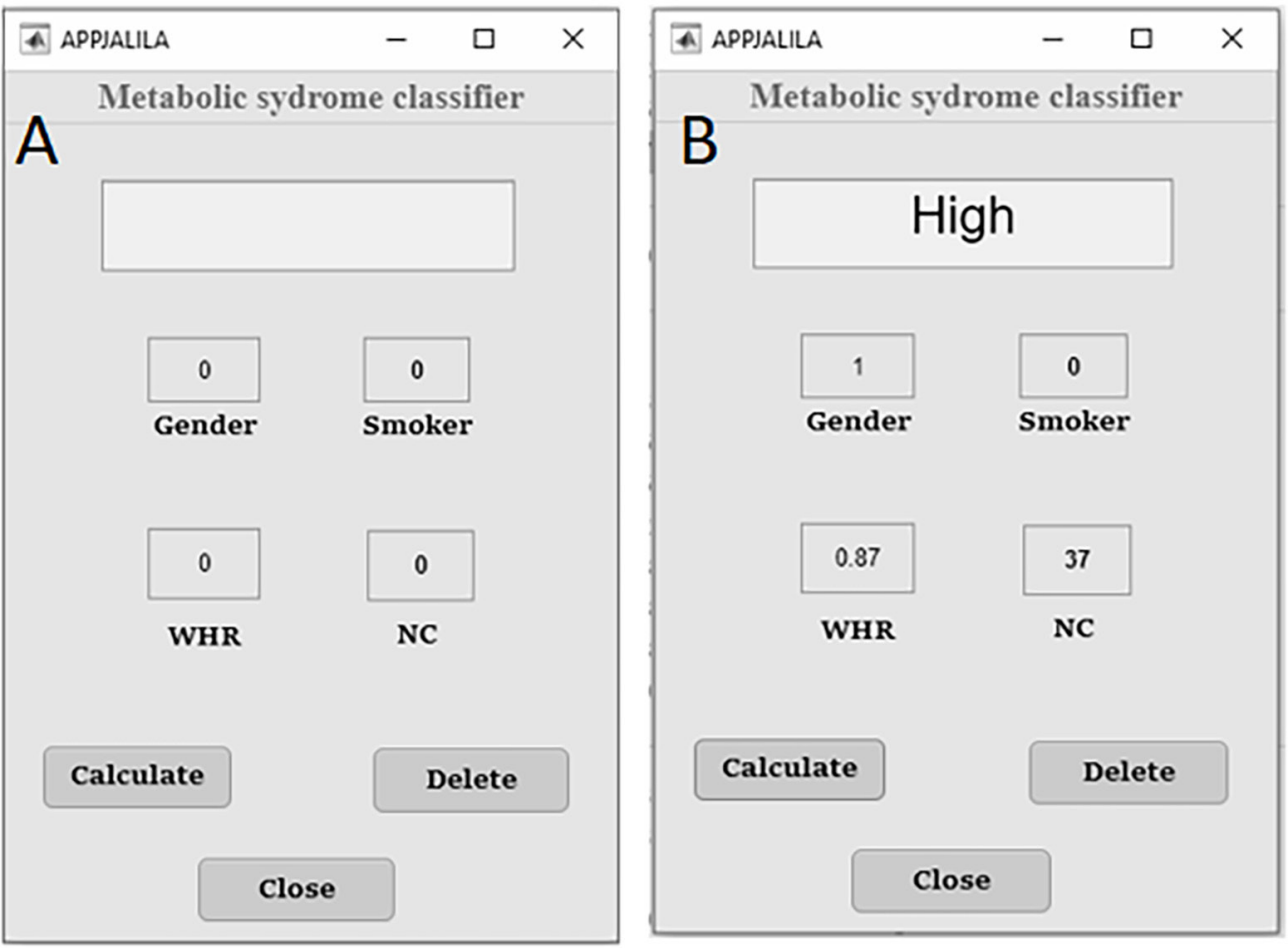
User interface layout (A) and example with input parameters (B).

The software is simple and easy to use. It has four fields for entering the patient’s clinical data and three buttons. These buttons are labeled as follows: calculate (provides the patient classification after analyzing the clinical data), clear (deletes the entered data on the screen), and close (terminates the operation of the software). The conclusion from the analysis of the algorithm is presented in the “Result” field and described as follows: high or normal for predicting MS risk in CKD patients, as shown in Figure 2.

## Discussion

Corroborating our results, several studies reported a higher incidence of MS in women with severe CKD and a higher prevalence in the elderly^19–25^. Age is considered a risk factor for both MS and CKD. In this study, the high prevalence of MS in elderly people can be described by functional limitations, an increasingly sedentary lifestyle, and reduced physical activity, as described in other reports^26^.

The results of our study reveal that abdominal obesity, altered blood pressure, and high blood glucose were associated with CKD. In addition, the main underlying diseases in the study population were SAH and DM. These findings confirm the results of studies in which central obesity was associated with CKD, regardless of general obesity and increased BMI^25,27,28^.

In MS, abdominal obesity is one of the main components responsible for insulin resistance, which in turn can lead to progressive loss of renal function^29^. This occurs because obesity directly affects hemodynamics and renal structure, as substantial evidence shows^30^.

Among CNCD, SAH and DM were reported as the main underlying diseases in our study. These pathologies are among the most prevalent risk factors for the development of CKD and are responsible for most cases. In Brazil, according to the Census of Hemodialysis Centers, hypertensive nephropathy (34%) and diabetes (31%) are the main underlying diseases in patients undergoing hemodialysis in 2019^31^.

In this study, MS (according to the IDF definition) is associated with an increased risk of CKD^18^. These findings call for greater attention to policies and interventions, such as lifestyle changes, that should aim to reduce the prevalence of MS and its adverse outcomes. The literature suggests that efforts to raise awareness of prevention strategies should start early, when any of the constituent components of MS is present^19^.

Thus, public health strategies are important for the prevention of CNCD in general. To this end, the Strategic Action Plan for Confronting CNCDs 2011–2022 was launched to promote the development of public policies. Itaimed at the prevention and control of CNCDs and their grievances in order to reduce the premature mortality rates (30 to 69 years) by 2% per year and reduce the prevalence of their risk factors^32^.

In this study, MS had a higher prevalence in the severe CKD group. It is already known that MS negatively impacts the progression of CKD. Therefore, MS and its associated factors need to be identified early. However, a disadvantage in MS evaluation is the use of invasive variables that are included in all criteria for MS diagnosis^18,33^.

In developing countries, as in Brazil, the population’s difficulty in accessing primary health care services, specialized consultations, and complementary exams contribute to the underreporting of CNCD, including MS and CKD. Therefore, the development of complementary, cost-effective and easy-to-use diagnostic methods is needed to facilitate patient access to early diagnosis^14–16^.

In this sense, the use of additive manufacturing techniques appears to be an instrument to assist in the development of new low-cost screening methods without the use of invasive variables^34^. Therefore, one of the proposals of this study was the development of a software with these characteristics, such as the KNN algorithm, which can be used in the evaluation of CDK patients with MS.

The KNN method performs binary classification by not only giving the outputs, when there is pathology and when there is no pathology, but also performing the expected classification based on the evaluation of the collected data. Several studies use the KNN method to classify groups for application to biological data, with satisfactory results, indicating that it is an efficient method for the present work^21,34^.

With the demand for methods that can facilitate diagnoses and optimize the care of healthcare professionals, several studies have been developed to address the application of ML in healthcare. These include the following: prediction of cardiovascular disease over a 10-year period^35^; predictive models for undiagnosed DM^36^; and Parkinson’s disease diagnosis from patient writing, by using image processing techniques^37^.

In the present study, based on the evaluation metrics of the chosen classifier and the area under the ROC curve (AUC = 0.79), KNN was found to be a good predictor of MS tracking in clinical practice. In Rosa’s study evaluating the prediction of metabolic syndrome in antiretroviral users, the KNN algorithm also proved to be a good predictor by providing AUC = 0.78, a result similar to ours^38^.

This result can be attributed to input parameters such as NC and WHR, for example. This statement is contradicted by several findings in the literature, which report the importance of anthropometric variables for clinical practice by associating NC^37,39^ and WHR^40,41^ as good predictors of MS.

Similarly, smoking is an established indicator in the literature for cardiometabolic risk analysis^42,43^. Smoking remains the leading cause of preventable death worldwide and a crucial factor for the development of CNCDs such as cancer, cardiovascular, and pulmonary diseases. Gender, in turn, has been associated with MS and CKD, with higher prevalence among women^43,44^.

The advantages of the KNN are: its simplicity of implementation, its very effective performance in different situations and areas (engineering, health, education, among others), its ease of interpretation, and its ideal suitability for small or medium-sized databases. Furthermore, it directly produces the decision rule without estimating the densities that are conditioned on the classes, making it a good choice for classification problems where closely related patterns in the feature space may belong to the same class^21^.

Thus, the KNN algorithm proved to be an attractive tool that can be implemented in environments with few resources. It can also support the clinical management of MS in the general population, especially in the CKD population, given the association between the number of MS components and the progression of CKD. In addition, it can have a positive impact on the quality of life of this population.

However, some limitations were found throughout the course of the study. One of the initial limitations was due to the COVID-19 pandemic, which made data collection difficult since the study population was considered at risk for the coronavirus. The number of variables can be seen as another limiting factor, because it is believed that using more input variables can increase the specificity of the classifier algorithm. Another additional limitation can be the inclusion of competitive risks, where patients may suffer from other types of events that prevent the observation of the event under study. Furthermore, as this was a cross-sectional study, it was not possible to continue monitoring patients over time, and it was not possible to establish a cause-effect relationship.

This contributed to a limitation of the sample, as the results found should only be considered for the population in question. Thus, as a recommendation for future investigations in this topic, we emphasize the importance of developing the classifier algorithm at this time. For this, a more robust sample can be used by expanding it to other populations with other associated comorbidities, by increasing the number of input variables, and by developing a longitudinal study. Also, a validation study of the implemented software, an incorporation of additional statistical tests, and a comparison of different algorithms can be provided, with or without the same proposed methods.

## Conclusion

In summary, there was a significant prevalence of MS in the CKD population, demonstrating the importance of early detection of this syndrome. This occurs because of the fact that MS involves a series of cardiometabolic factors, and the more components present, the greater the risk of CKD progression. The KNN classifier algorithm can be used as a screening method with high sensitivity and low cost. In addition, it can be used to screen for MS in primary healthcare units and low-resource settings by contributing to the early detection of MS in the general population, especially in CKD. The use of the classifier can help health professionals in decision making by contributing to preventive healthcare, especially in CKD, to reduce treatment costs and even avoid negative outcomes and early death.

Considering its important usefulness for the public health field, another possible area of future research could be to carry out larger studies with the KNN classifier and its variants. Also, other low-cost parameters could be used to predict the risk of harmful clinical conditions in groups with and no risk of chronic non-communicable diseases.
