# Supplementary material for: Prediction of metabolic syndrome and its associated risk factors in patients with chronic kidney disease using machine learning techniques
Source: J Bras Nefrol. 2024 Aug 9;46(4):e20230135. doi: 10.1590/2175-8239-JBN-2023-0135en (PMC11318987; doi:10.1590/2175-8239-JBN-2023-0135en)
Supplement: Translation

# Translation: Predição de síndrome metabólica e seus fatores de risco associados em pacientes com doença renal crônica utilizando técnicas de *machine learning*

**DOI:** 10.1590/2175-8239-JBN-2023-0135pt

**Authors:** Jalila Andréa Sampaio Bittencourt, Carlos Magno Sousa, Ewaldo Eder Carvalho Santana, Yuri Armin Crispim de Moraes, Erika Cristina Ribeiro de Lima Carneiro, Ariadna Jansen Campos Fontes, Lucas Almeida das Chagas, Naruna Aritana Costa Melo, Cindy Lima Pereira, Margareth Costa Penha, Nilviane Pires, Edward Araujo, Allan Kardec Duailibe Barros, Maria do Desterro Soares Brandão Nascimento

**Affiliations:** 1 Universidade Federal do Maranhão, Departamento de Engenharia Eletrônica, Laboratório de Processamento da Informação Biológica, São Luiz, MA, Brasil.; 2 Universidade Federal do Maranhão, Departamento de Ciência da Computação, Laboratório de Aquisição e Processamento de Sinais, São Luiz, MA, Brasil.; 3 Universidade Federal do Maranhão, Hospital Universitário, Centro de Prevenção de Doenças Renais, São Luiz, MA, Brasil.; 4 Universidade Federal do Maranhão, Centro de Ciências Biológicas e da Saúde, Laboratório de Imunofisiologia, São Luiz, MA, Brasil.; 5 Universidade Federal de São Paulo, Escola Paulista de Medicina, Departamento de Obstetrícia, São Paulo, SP, Brasil.; 6 Universidade Federal do Maranhão, Laboratório de Ciências Biológicas, Laboratório de Genética e Biologia Molecular, São Luiz, MA, Brasil.; 7 Universidade Ceuma, Departamento de Biomedicina, Laboratório de Ciências Biomédicas, São Luiz, MA, Brasil.; 8 Universidade Federal do Maranhão, Departamento de Medicina, Programa de Pós-Graduação em Saúde do Adulto, São Luiz, MA, Brasil.

**Keywords:** Inteligência Artificial, Insuficiência Renal Crônica, Aprendizado de Máquina, Síndrome Metabólica

## Abstract

**Introdução:**

A doença renal crônica (DRC) e a síndrome metabólica (SM) são reconhecidas como problemas de saúde pública relacionados ao excesso de peso e a fatores cardiometabólicos. O objetivo deste estudo foi desenvolver um modelo para prever a SM em pessoas com DRC.

**Métodos:**

Este foi um estudo transversal prospectivo de pacientes de um centro de referência em São Luís, MA, Brasil. A amostra incluiu voluntários adultos classificados de acordo com a presença de DRC leve ou grave. Para o rastreamento da SM, o algoritmo de classificação k-nearest neighbors (KNN) foi utilizado com os seguintes dados: sexo, tabagismo, circunferência do pescoço e relação cintura-quadril. Os resultados foram considerados significativos com p < 0,05.

**Resultados:**

Foram avaliados 196 pacientes adultos com média de idade de 44,73 anos, 71,9% do sexo feminino, 69,4% com sobrepeso e 12,24% com DRC. Desses últimos, 45,8% apresentaram SM, a maioria tinha até 3 componentes metabólicos alterados, e o grupo com DRC apresentou significância estatística em: circunferência da cintura, pressão arterial sistólica, pressão arterial diastólica e glicemia de jejum. O algoritmo KNN comprovou ser um bom preditor para a triagem de SM com acurácia e sensibilidade de 79% e especificidade de 80% (área sob a curva ROC – AUC = 0,79).

**Conclusão:**

O algoritmo KNN pode ser usado como um método de triagem de baixo custo para avaliar a presença de SM em pessoas com DRC.

## Introdução

As doenças crônicas não transmissíveis (DCNT) são atualmente reconhecidas como um dos principais problemas de saúde pública^1^. A Organização Mundial da Saúde (OMS) estima que as DCNTs sejam responsáveis por 71% dos 57 milhões de óbitos ocorridos mundialmente^2^. No Brasil, as DCNTs são responsáveis por 76,4% de todos os óbitos, com destaque para doenças do aparelho circulatório (28% dos óbitos), câncer (18%), diabetes mellitus (5%) e doenças respiratórias (6%)^3^.

Dentre as DCNT, destaca-se a doença renal crônica (DRC), que se caracteriza pela alteração da função renal. É definida como uma anormalidade na estrutura ou função renal que está presente há mais de três meses e traz implicações para a saúde. Essas anormalidades podem ser representadas por uma taxa de filtração glomerular (TFG) reduzida <60 ml/min/1,73 m ou pela presença de um ou mais marcadores de injúria renal^4,5^.

A prevalência de DRC ainda é desconhecida em muitos países^6^. Entretanto, tem aumentado, principalmente como resultado do aumento da incidência de obesidade, diabetes e hipertensão. Além disso, a função renal é altamente suscetível a alterações relacionadas à idade, com uma incidência significativamente maior em pacientes de meia-idade e idosos^7,8^.

Pessoas com DRC tendem a apresentar doenças cardiovasculares (DCV)^8^. A DCV é a principal causa de óbito em pacientes com doença renal crônica e está associada à progressão acelerada da DRC. Esses achados corroboram a ideia de que a presença de fatores de risco cardiometabólicos (FRC) e a função renal prejudicada podem aumentar os riscos relacionados à doença renal^9^.

Além disso, a síndrome metabólica (SM) é considerada um agrupamento de fatores de risco inter-relacionados que duplica o risco de DCV em 5 a 10 anos^10^. Essa patologia é descrita como um conjunto de FRC, que geralmente estão relacionados ao desenvolvimento de resistência à insulina e acúmulo de gordura. Esses fatores de risco incluem hipertensão arterial, hipertrigliceridemia, dislipidemia, hiperglicemia e obesidade central^11^.

Os indivíduos com DNTs geram custos financeiros elevados ao sistema público de saúde, pois requerem tratamento para o controle dessas doenças, especialmente no caso de SM e DRC. Os pacientes com DRC em estágio terminal, estágio G5 (TFG <15 mL/min/1,73 m^2^) apresentam insuficiência renal grave que leva à perda completa da função renal. Nesse estágio, as opções terapêuticas são as terapias renais substitutivas (TRS), como métodos artificiais de purificação do sangue (diálise peritoneal ou hemodiálise) ou transplante renal^4^. No Brasil, a TRS é considerada o principal tratamento e também exige custos mais elevados para os serviços de saúde^12^. Portanto, a detecção precoce dessas patologias pode retardar complicações e apoiar o uso de intervenções adequadas, como testes de triagem em grupos de alto risco^13^.

Nesse caso, algumas técnicas de análise de dados parecem ser boas soluções que fornecem previsões mais precisas sobre a saúde do indivíduo^14^. Assim, o uso de técnicas de *machine learning* (ML) parece ser um instrumento para ajudar a desenvolver e melhorar novos métodos de diagnóstico e/ou triagem^15,16^.

Portanto, a importância do estudo para a predição de SM na população com DRC é evidente, uma vez que essa condição leva a estágios mais avançados e a um maior risco de óbito por eventos cardiovasculares nessa população. Dessa forma, mesmo que os pacientes com DRC apresentem poucos fatores de risco para SM, medidas preventivas devem ser tomadas para evitar problemas e desfechos negativos, como óbito precoce.

Assim, dada a magnitude da SM e da DRC e as complicações a elas relacionadas, deve-se, portanto, envidar esforços para viabilizar estudos destinados ao diagnóstico precoce dessas patologias. Além disso, este estudo tem como objetivo desenvolver um modelo para prever o risco de SM e os fatores de risco associados em pessoas com DRC.

## Métodos

Este foi um estudo transversal prospectivo de pacientes de ambos os sexos do Centro de Referência em Nefrologia em São Luís, MA, Brasil, entre janeiro de 2018 e julho de 2020. A amostra consistiu em 196 voluntários classificados de acordo com seu estado de saúde A DRC foi determinada por uma taxa de filtração glomerular <60 mL/min^4^, para DRC leve (acima desse valor) ou DRC grave. A TFG foi determinada pela medição da creatinina sérica. Para calcular a taxa de filtração glomerular estimada, foi utilizada a equação *Chronic Kidney Disease Epidemiology Collaboration* (CKD-EPI)^17^.

A suspeita diagnóstica de SM foi definida conforme proposto pela *International Diabetes Federation* (IDF)^18^ considerando a presença de alterações na circunferência da cintura (≥ 90 cm para homens e ≥ 80 cm para mulheres) como um fator obrigatório, além de outros dois componentes alterados. Esses componentes podem ser: triglicerídeos ≥ 150 mg/dL ou tratamento para dislipidemia; colesterol HDL < 40 mg/dL para homens e < 50 mg/dL para mulheres ou tratamento para dislipidemia; pressão arterial sistólica (PAS) ≥ 130 mmHg, pressão arterial diastólica (PAD) ≥ 85 mmHg ou uso de medicação anti-hipertensiva; e glicemia de jejum ≥ 100 mg/dL ou diagnóstico prévio de diabetes mellitus^19^.

A coleta de dados foi realizada em duas etapas. Primeiro, os voluntários foram recrutados no Centro de Nefrologia. Aqueles que concordaram em participar do estudo assinaram voluntariamente o Termo de Consentimento Livre e Esclarecido (TCLE). Em seguida, foram entrevistados por meio de um questionário semiestruturado, seguido de uma avaliação antropométrica e hemodinâmica. Os exames laboratoriais foram agendados para o dia seguinte.

Foram avaliados dados antropométricos, bioquímicos, hemodinâmicos e de estilo de vida. O questionário semiestruturado considerou características sociodemográficas, estilo de vida e histórico pessoal autorrelatado, como hipertensão e diabetes mellitus. As variáveis antropométricas foram realizadas em duplicata, e as médias foram utilizadas para a análise dos dados. Todas as variáveis foram medidas de acordo com protocolos já consolidados na literatura^20^. Essas variáveis foram: peso, altura, circunferência do braço (CB), circunferência da cintura (CC), circunferência do quadril (CQ), circunferência do pescoço (CP) e circunferência da panturrilha (CPa).

Índices antropométricos: relação cintura-quadril (RCQ), relação cintura-estatura (RCE) e índice de massa corporal (IMC) foram utilizados para determinar o estado nutricional e foram baseados nos pontos de corte da OMS. A CC foi estimada considerando os pontos de corte para a população sul-americana, com valores de ≥90 cm para homens e ≥80 cm para mulheres^19^.

Somente o algoritmo de classificação *k-nearest neighbors* (KNN), um método de ML supervisionado, foi utilizado para o rastreamento da SM. É fácil de implementar, adaptável e simples de programar. Todas essas vantagens tiveram um impacto positivo em nossa escolha, pois o KNN por si só atendeu às nossas demandas^21^.

O KNN utiliza os dados mais próximos e realiza uma segmentação dos resultados mais próximos com base na métrica selecionada, considerando uma margem de erro limitada. Nesse algoritmo, o conjunto de dados é preparado pela remoção de dados perdidos e pela normalização das características, o que é conhecido como fase de pré-processamento. Os dados são então divididos aleatoriamente em dois conjuntos diferentes: o conjunto de treinamento e o conjunto de teste. Essa técnica garante uma representação adequada dos padrões para treinamento e uma avaliação robusta de desempenho^21^.

Portanto, o banco de dados foi dividido em dois grupos: 80% da amostra foi para treinamento e 20% para teste. Com base nas informações clínicas do paciente (sexo, tabagismo, RCQ e CPa), o algoritmo de classificação foi capaz de distinguir indivíduos com e sem SM. O método de classificação foi construído com o uso do software MATLAB® versão R2021a (MathWorks Inc, Natick, MD, EUA).

Para o arquivo de dados e análise estatística, foi utilizado o software SPSS® versão 25 (SPSS Inc., Chicago, IL, EUA). O teste de Kolmogorov-Smirnov foi usado para analisar a normalidade dos dados. As variáveis que foram consideradas com distribuição normal foram analisadas por meio do teste t de Student. As demais foram analisadas com o teste U de Mann-Whitney, e a curva ROC (*Receiver Operating Characteristics*) foi utilizada para a avaliação do algoritmo de classificação. Além disso, os resultados foram considerados estatisticamente significativos quando p < 0,05. Todos os valores da análise estatística estão descritos nas tabelas da seção Resultados.

Este estudo faz parte de um projeto maior (“guarda-chuva” ou “raiz”), intitulado *“Prediction of Chronic Kidney Disease Using Artificial Neural Networks”* (Predição de Doença Renal Crônica Utilizando Redes Neurais Artificiais). Também foi aprovado pelo Comitê de Ética e Pesquisa da Universidade Federal do Maranhão, de acordo com o parecer CAAE número 67030517.5.0000.5087. Além disso, todos os participantes assinaram o termo de consentimento livre e esclarecido. Vale ressaltar que este trabalho utiliza dados populacionais originais gerados exclusivamente para esta pesquisa, além de fornecer novos dados para o projeto guarda-chuva. Ademais, é importante mencionar que tanto as redes neurais quanto o KNN são modelos computacionais de inteligência artificial que trabalham com processamento de dados para classificação e predição.

## Resultados

Foram avaliados 196 pacientes adultos com média de idade de 44,73 ± 15,96 anos, dos quais 71,9% (n = 141) eram do sexo feminino e 79,1% (n = 155) se identificaram como não brancos. Além disso, 65,8% (n = 129) dos pacientes relataram não praticar atividade física, 29,1% (n = 57) consumiam álcool e 3,6% (n = 7) fumavam. Em relação às doenças subjacentes, 31,1% (n = 61) relataram ter hipertensão arterial sistêmica (HAS) e 29,6% (n = 58) apresentavam diabetes mellitus (Tabela 1). A análise dos dados antropométricos revelou que mais da metade dos indivíduos estavam com sobrepeso (69,4%), com IMC médio de 27,44 ± 5,10 kg/m^2^ (Tabelas 1 e 2).

**Tabela 1:** Características sociodemográficas e de estilo de vida da amostra

Variáveis	Categoria	N	%
Cor da pele^*^	Branca	41	20,9%
	Não branca	155	79,1%
Sexo^*^	Feminino	141	71,9%
	Masculino	55	28,1%
Status nutricional (IMC)^*^	Eutrófico	60	30,6%
	Sobrepeso	136	69,4%
Tabagismo^*^	Não	189	96,4%
	Sim	7	3,6%
Alcoolismo^*^	Não	139	70,9%
	Sim	57	29,1%
Prática de atividade física^*^	Não	129	65,8%
	Sim	67	34,2%
Presença de DRC^*^	Não	172	87,8%
	Sim	24	12,2%
Presença de SM^*^	Não	150	76,5%
	Sim	46	23,5%
DM autorrelatado^*^	Não	138	70,4%
	Sim	58	29,6%
HAS autorrelatada^*^	Não	135	68,9%
	Sim	61	31,1%

Abreviações – IMC: índice de massa corporal; DRC: doença renal crônica; SM: síndrome metabólica; DM: diabetes mellitus; HAS: hipertensão arterial sistêmica. Notas – *valores descritos como porcentagem (%) e frequência (n).

**Tabela 2:** características antropométricas e hemodinâmicas da amostra

Variáveis	Médias
Idade (anos)^†^	44,73 ± 15,96
Altura (m)^†^	1,58 ± 0,08
Peso corporal (kg)^†^	68,63 ± 13,79
CC (cm)^†^	87,22 ± 12,95
CB (cm)^†^	30,46 ± 4,08
CP (cm)^†^	35,16 ± 3,53
CPa (cm)^†^	35,66 ± 3,63
IMC (kg/m^2^)^†^	27,44 ± 5,10
RCE^†^	0,55 ± 0,08
RCQ†	0,85 ± 0,08
PAS^†^	124,06 ± 23,99
PAD^†^	77,37 ± 12,06

Abreviações – CC: circunferência da cintura; CB: circunferência do braço; CP: circunferência do pescoço; CPa: circunferência da panturrilha; IMC: índice de massa corporal; RCE: relação cintura/estatura; RCQ: relação cintura:quadril; PAS: pressão arterial sistólica; PAD: pressão arterial diastólica; m: metros; cm: centímetros. Notas – †Teste U de Mann-Whitney; Dados descritos como média ± (desvio padrão).

A Tabela 3 mostra as características gerais da amostra, que foi estratificada de acordo com a presença de DRC leve e grave. Nessa Tabela, os pacientes com DRC grave apresentaram valores significativamente mais elevados de pressão arterial sistólica e diastólica, idade, CP, glicemia de jejum e ureia (p < 0,05) do que aqueles com DRC leve. Da mesma forma, as variáveis indicadoras de obesidade (CC, IMC, RCQ, RCE) também apresentaram uma prevalência maior no grupo de DRC grave.

**Tabela 3:** Características antropométricas, hemodinâmicas e laboratoriais da amostra, estratificadas pela presença de doença renal crônica (DRC)

Variáveis	DRC leve	DRC grave	Valor de p
	(n = 172)	(n = 24)	
Idade (anos)^†^	40,5 (29,25 – 54)	60 (51,25 – 68)	<0,001
Altura (m)^†^	1,57 (1,51 – 1,63)	1,61 (1,55 – 1,68)	0,153
Peso corporal (kg)^†^	66,2 (58,8 – 76,57)	70,45 (64,17 – 80,45)	0,090
CC (cm)^†^	86,16 ± 12,79	94,85 ± 11,7	0,002
CB (cm)^†^	30 (28 – 33,3)	30 (28,12 – 31,87)	0,816
CP (cm)^†^	34,9 (32 – 38)	36,75 (34 – 39,75)	0,039
CPa (cm)^†^	35,5 (33 – 38)	34,25 (32 – 36,75)	0,196
IMC (kg/m^2^)^†^	26,54 (23,71 – 29,91)	27,67 (25,58 – 31,42)	0,285
RCQ^†b^	0,84 ± 0,08	0,93 ± 0,08	<0,001
RCE^†^	0,54 ± 0,08	0,59 ± 0,07	0,012
PAS^†^	117,5 (107 – 131)	134 (123,25 – 165,5)	<0,001
PAD^†^	75 (70 – 82)	86,5 (76,25 – 91)	0,019
CT^†^	178,12 ± 45,39	163,13 ± 44,78	0,131
Triglicerídeos^†^	108 (75,25 – 164,75)	128 (84,25 – 174,5)	0,216
Colesterol HDL^†^	44 (38 – 54)	43 (34 – 53,75)	0,575
Colesterol LDL^†^	105,73 ± 36,41	89,80 ± 41,89	0,050
Glicose no sangue^†^	79,00 (77,50 – 87,50)	81,00 (75,00 – 88,00)	0,001
Ureia^†^	24,5 (20,25 – 31)	54,5 (39,25 – 79)	<0,001

Abreviações – IMC: índice de massa corporal; CC: circunferência da cintura; CB: circunferência do braço; CPa: circunferência da panturrilha; RCQ: relação cintura:quadril; RCE: relação cintura/estatura; PAS: pressão arterial sistólica; PAD: pressão arterial diastólica; CT: colesterol total; TFG: taxa de filtração glomerular; m: metros; cm: centímetros. Notas – †Teste U de Mann-Whitney; Valores descritos como média/mediana ± DP (desvio padrão).

Para fins clínicos, epidemiológicos, didáticos e conceituais, a DRC é classificada em seis estágios funcionais, de acordo com o grau de função renal do paciente, baseado na taxa de filtração glomerular, variando de condição normal/alta a diálise ou transplante. O KDIGO (*Kidney Disease: Improving Global Outcomes*) orienta a estimativa da TFG a partir da creatinina sérica como o melhor método para diagnosticar, classificar e monitorar a progressão da DRC^4^. A TFG foi categorizada de G1 a G5, conforme demonstrado na Tabela 4.

**Tabela 4:** Estadiamento e classificação da doença renal crônica (DRC) na amostra (n = 196)

Estágios	Classificação		Frequência*	
G1	TFG >90	(normal ou aumentada)	117	41,1%
G2	TFG 60–89	(levemente reduzida)	55	19,3%
G3a	TFG 45–59	(redução leve a moderada)	10	3,5%
G3b	TFG 30–44	(redução moderada a grave)	5	1,8%
G4	TFG 15–29	(gravemente reduzida)	5	1,8%
G5	TFG <15	(insuficiência renal)	4	1,4%

Abreviação: TFG: taxa de filtração glomerular. Nota – *valores descritos em frequência (n) e porcentagem (%).

Quanto ao estadiamento e classificação da DRC, a Tabela 4 mostra que uma grande parte da amostra está nos estágios iniciais (G1 e G2), todos do grupo DRC leve. Em contraste, somente 1,4% (n = 4) está em estágio terminal e requer algum tipo de terapia renal substitutiva.

A Tabela 5 mostra os valores de prevalência dos fatores de risco para síndrome metabólica estratificados pela presença de DRC leve ou grave. A análise dos dados clínicos mostrou que, em valores percentuais, todos os componentes alterados da SM se mostraram prevalentes no grupo DRC grave, com exceção do colesterol total (CT). Da amostra investigada, 45,8% dos pacientes com DRC grave apresentaram SM, e a maioria tinha até três componentes metabólicos alterados.

**Tabela 5:** Prevalência de síndrome metabólica e seus componentes na amostra

Variáveis	Categoria	DRC leve		DRC grave		Valor de p
		N	%	N	%	
SM^*^	Não	137	79,70%	13	54,20%	0,006
	Sim	35	20,30%	11	45,80%
CC^*^	Normal	68	39,50%	6	25,00%	0,169
	Alterada	104	60,50%	18	75,00%
PA^*^	Normal	113	65,70%	7	29,20%	0,001
	Alterada	59	34,30%	17	70,80%
CT^*^	Normal	108	62,80%	17	70,80%	0,443
	Alterada	64	37,20%	7	29,20%
TG^*^	Normal	121	70,30%	15	62,50%	0,434
	Alterada	51	29,70%	9	37,50%
HDL^*^	Não	75	43,60%	10	41,70%	0,858
	Sim	97	56,40%	14	58,30%
GJ^*^	Normal	149	86,60%	15	62,50%	0,003
	Alterada	23	13,40%	9	37,50%

Abreviações – CC: circunferência da cintura; PA: pressão arterial; CT elevado: colesterol total elevado; TG: triglicerídeos; HDL: lipoproteína de alta densidade; GJ: glicemia de jejum. Nota – *Teste qui-quadrado.

Ao observarmos a tabela estratificada pela presença de DRC (Tabela 3), o grupo de indivíduos com problemas renais apresentou valores médios/medianos mais elevados em todos os parâmetros em comparação com indivíduos saudáveis, com significância estatística na CC, pressão arterial sistólica, pressão arterial diastólica e glicemia de jejum.

Com relação ao software desenvolvido por meio de ML, o algoritmo implementado foi o KNN com as seguintes entradas: sexo, tabagismo, RCQ e CP. O desenvolvimento do algoritmo foi rotulado com base nos componentes da SM de acordo com os critérios da IDF^18,19^.

Não há uma proporção fixa que funcione em todos os cenários do algoritmo KNN. O banco de dados foi dividido em dois grupos: 80% da amostra foi alocada para treinamento e 20% para teste. Isso foi feito considerando o fato de que, com conjuntos de dados muito grandes, pode ser viável utilizar proporções como 70/30 ou mesmo 90/10^22^. Todas essas divisões foram testadas, e a que apresentou a melhor resposta foi 80/20, por se tratar de um banco de dados de tamanho médio. O KNN teve 79% de acurácia e sensibilidade e especificidade de 80%.

A Figura 1 mostra a representação gráfica da curva ROC (área sob a curva ROC – AUC = 0,79) gerada pela plotagem da sensibilidade (taxa de verdadeiros positivos) no eixo y versus a especificidade (taxa de falsos positivos) no eixo x. Assim, o KNN comprovou ser um bom classificador para prever a SM. Para que um teste diagnóstico seja considerado preciso, uma curva no triângulo superior esquerdo deve estar acima da linha de referência. Quanto mais próxima essa curva estiver desse canto, ou seja, mais próxima de 1, melhor o desempenho do método^23,24^.

**Figura 1:** A área sob a curva ROC demonstra o poder discriminatório do algoritmo de *k-nearest neighbors* na predição da síndrome metabólica no conjunto de teste.

Nesta primeira etapa do estudo, decidimos utilizar esses parâmetros antropométricos por serem de baixo custo, fáceis de usar e já recomendados na literatura. No entanto, acredita-se que o uso de mais variáveis de entrada possa aumentar a especificidade do algoritmo de classificação.

O software é simples e fácil de usar. Possui quatro campos para inserção dos dados clínicos do paciente e três botões. Esses botões são rotulados da seguinte forma: calcular (fornece a classificação do paciente após a análise dos dados clínicos), limpar (exclui os dados inseridos na tela) e fechar (encerra a operação do software). A conclusão da análise do algoritmo é apresentada no campo “Resultado” e descrita como segue: alta ou normal para predição do risco de SM em pacientes com DRC, conforme mostrado na Figura 2.

**Figura 2:** Layout da interface do usuário (A) e exemplo com parâmetros de entrada (B).

## Discussão

Corroborando nossos resultados, diversos estudos relataram maior incidência de SM em mulheres com DRC grave e maior prevalência em idosos^19–25^. A idade é considerada um fator de risco tanto para a SM quanto para a DRC. Neste estudo, a alta prevalência de SM em pessoas idosas pode ser descrita por limitações funcionais, um estilo de vida cada vez mais sedentário e atividade física reduzida, conforme descrito em outros relatos^26^.

Os resultados de nosso estudo revelam que obesidade abdominal, pressão arterial alterada e glicemia elevada foram associadas à DRC. Além disso, as principais doenças subjacentes na população do estudo foram HAS e DM. Esses achados confirmam os resultados de estudos nos quais a obesidade central foi associada à DRC, independentemente da obesidade geral e do aumento do IMC^25,27,28^.

Na SM, a obesidade abdominal é um dos principais componentes responsáveis pela resistência à insulina, que, por sua vez, pode levar à perda progressiva da função renal^29^. Isso ocorre porque a obesidade afeta diretamente a hemodinâmica e a estrutura renal, como demonstram evidências substanciais^30^.

Entre as DCNT, a HAS e o DM foram relatados como as principais doenças subjacentes em nosso estudo. Essas patologias estão entre os fatores de risco mais prevalentes para o desenvolvimento da DRC e são responsáveis pela maioria dos casos. No Brasil, de acordo com o Censo dos Centros de Hemodiálise, a nefropatia hipertensiva (34%) e o diabetes (31%) são as principais doenças subjacentes em pacientes submetidos à hemodiálise em 2019^31^.

Neste estudo, a SM (de acordo com a definição da IDF) está associada a um risco aumentado de DRC^18^. Esses resultados exigem maior atenção às políticas e intervenções, como mudanças no estilo de vida, que devem ter como objetivo reduzir a prevalência da SM e seus desfechos adversos. A literatura sugere que os esforços para aumentar a conscientização sobre estratégias de prevenção devem começar cedo, quando qualquer um dos componentes constituintes da SM estiver presente^19^.

Assim, as estratégias de saúde pública são importantes para a prevenção de DCNT em geral. Para esse fim, foi lançado o Plano de Ações Estratégicas para o Enfrentamento das DCNTs 2011–2022 para promover o desenvolvimento de políticas públicas. O objetivo era a prevenção e o controle das DCNTs e seus agravos, a fim de reduzir as taxas de mortalidade prematura (30 a 69 anos) em 2% ao ano e reduzir a prevalência de seus fatores de risco^32^.

Neste estudo, a SM teve maior prevalência no grupo com DRC grave. Já é sabido que a SM afeta negativamente a progressão da DRC. Portanto, a SM e seus fatores associados precisam ser identificados precocemente. Entretanto, uma desvantagem na avaliação da SM é o uso de variáveis invasivas que estão incluídas em todos os critérios para o diagnóstico da SM^18,33^.

Em países em desenvolvimento, como no Brasil, a dificuldade da população em acessar serviços de atenção primária à saúde, consultas especializadas e exames complementares contribui para a subnotificação de DCNT, incluindo SM e DRC. Portanto, é necessário o desenvolvimento de métodos diagnósticos complementares, com custo-benefício e fáceis de usar para facilitar o acesso dos pacientes ao diagnóstico precoce^14–16^.

Nesse sentido, o uso de técnicas de manufatura aditiva parece ser um instrumento para auxiliar no desenvolvimento de novos métodos de triagem de baixo custo sem a utilização de variáveis invasivas^34^. Sendo assim, uma das propostas deste estudo foi o desenvolvimento de um software com essas características, como o algoritmo KNN, que pode ser usado na avaliação de pacientes com DRC e SM.

O método KNN realiza a classificação binária não apenas fornecendo os resultados, quando há ou não patologia, mas também realizando a classificação esperada com base na avaliação dos dados coletados. Diversos estudos utilizam o método KNN para classificar grupos para aplicação em dados biológicos, com resultados satisfatórios, indicando que esse é um método eficiente para o presente trabalho^21,34^.

Com a demanda por métodos que possam facilitar os diagnósticos e otimizar o atendimento dos profissionais de saúde, diversos estudos têm sido desenvolvidos para abordar a aplicação do ML na área da saúde. Entre eles estão: previsão de doenças cardiovasculares em um período de 10 anos^35^; modelos preditivos para o DM não diagnosticado^36^; e diagnóstico da doença de Parkinson a partir da escrita do paciente, utilizando técnicas de processamento de imagens^37^.

No presente estudo, com base nas métricas de avaliação do classificador escolhido e na área sob a curva ROC (AUC = 0,79), o KNN foi considerado um bom preditor de rastreamento de SM na prática clínica. No estudo de Rosa, que avaliou a predição da síndrome metabólica em usuários de antirretrovirais, o algoritmo KNN também demonstrou ser um bom preditor, fornecendo AUC = 0,78, um resultado semelhante ao nosso^38^.

Esse resultado pode ser atribuído a parâmetros de entrada, como CP e RCQ, por exemplo. Essa afirmação é contrariada por diversos achados na literatura, que relatam a importância das variáveis antropométricas para a prática clínica, associando a CP^37,39^ e a RCQ^40,41^ como bons preditores de SM.

De forma semelhante, o tabagismo é um indicador estabelecido na literatura para a análise de risco cardiometabólico^42,43^. O tabagismo permanece como a principal causa mundial de óbito evitável e um fator crucial para o desenvolvimento de DCNTs, como câncer, doenças cardiovasculares e pulmonares. O sexo, por sua vez, tem sido associado à SM e à DRC, com maior prevalência entre mulheres^43,44^.

As vantagens do KNN são: sua simplicidade de implementação, seu desempenho muito eficaz em diferentes situações e áreas (engenharia, saúde, educação, entre outras), sua facilidade de interpretação e sua adequação ideal para bancos de dados de tamanho pequeno ou médio. Além disso, ele produz diretamente a regra de decisão sem estimar as densidades que estão condicionadas às classes, o que o torna uma boa opção para problemas de classificação nos quais padrões estreitamente relacionados no espaço de características podem pertencer à mesma classe^21^.

Assim, o algoritmo KNN mostrou-se uma ferramenta atrativa que pode ser implementada em ambientes com recursos limitados. Também pode apoiar o manejo clínico da SM na população em geral, especialmente na população com DRC, dada a associação entre o número de componentes da SM e a progressão da DRC. Além disso, pode ter um impacto positivo na qualidade de vida dessa população.

No entanto, algumas limitações foram encontradas durante o curso do estudo. Uma das limitações iniciais foi devido à pandemia da COVID-19, que dificultou a coleta de dados, uma vez que a população do estudo foi considerada de risco para o coronavírus. O número de variáveis pode ser visto como outro fator limitante, pois acredita-se que o uso de mais variáveis de entrada possa aumentar a especificidade do algoritmo de classificação. Outra limitação adicional pode ser a inclusão de riscos competitivos, em que os pacientes podem sofrer outros tipos de eventos que impedem a observação do evento em estudo. Além disso, por se tratar de um estudo transversal, não foi possível continuar monitorando os pacientes ao longo do tempo e não foi possível estabelecer uma relação de causa e efeito.

Isso contribuiu para uma limitação da amostra, pois os resultados encontrados devem ser considerados somente para a população em questão. Assim, como recomendação para futuras investigações sobre esse tópico, enfatizamos a importância de desenvolver o algoritmo de classificação neste momento. Para isso, é possível usar uma amostra mais robusta, expandindo-a para outras populações com outras comorbidades associadas, aumentando o número de variáveis de entrada e desenvolvendo um estudo longitudinal. Além disso, um estudo de validação do software implementado, uma incorporação de testes estatísticos adicionais e uma comparação de diferentes algoritmos podem ser fornecidos, com ou sem os mesmos métodos propostos.

## Conclusão

Em resumo, houve uma prevalência significativa de SM na população com DRC, demonstrando a importância da detecção precoce dessa síndrome. Isso ocorre devido ao fato de que a SM envolve uma série de fatores cardiometabólicos e, quanto mais componentes estiverem presentes, maior o risco de progressão da DRC. O algoritmo de classificação KNN pode ser usado como um método de triagem com alta sensibilidade e baixo custo. Além disso, pode ser utilizado para a triagem da SM em unidades de saúde primária e em ambientes com recursos limitados, contribuindo para a detecção precoce da SM na população em geral, especialmente na DRC. O uso do classificador pode auxiliar profissionais de saúde na tomada de decisões, contribuindo para o cuidado preventivo, especialmente na DRC, para reduzir os custos do tratamento e até mesmo evitar desfechos negativos e óbito precoce.

Considerando sua importante utilidade para o campo da saúde pública, outra possível área de pesquisas futuras pode ser a realização de estudos mais abrangentes com o classificador KNN e suas variantes. Além disso, outros parâmetros de baixo custo podem ser utilizados para prever o risco de condições clínicas prejudiciais em grupos com e sem risco de doenças crônicas não transmissíveis.
